# Functionalization of ^68^Ga-Radiolabeled Nanodiamonds with Octreotide Does Not Improve Tumor-Targeting Capabilities

**DOI:** 10.3390/ph17040514

**Published:** 2024-04-17

**Authors:** Thomas Wanek, Marco Raabe, Md Noor A Alam, Thomas Filip, Johann Stanek, Mathilde Loebsch, Christian Laube, Severin Mairinger, Tanja Weil, Claudia Kuntner

**Affiliations:** 1 Department of Biomedical Imaging and Image-Guided Therapy, Medical University of Vienna, 1090 Vienna, Austria; thomas.wanek@meduniwien.ac.at (T.W.);; 2 Preclinical Molecular Imaging, AIT Austrian Institute of Technology GmbH, 2444 Seibersdorf, Austria; thomas.filip@vetmeduni.ac.at (T.F.);; 3 Synthesis of Macromolecules, Max Planck Institute for Polymer Research, 55128 Mainz, Germany; 4 Institute of Inorganic Chemistry I, Ulm University, 89081 Ulm, Germany; 5 Institute of Atomic and Molecular Sciences, Academia Sinica, Taipei 115201, Taiwan; 6 Institute of Animal Breeding and Genetics, University of Veterinary Medicine, 1210 Vienna, Austria; 7 Core Facility Laboratory Animal Breeding and Husbandry (CFL), Medical University of Vienna, 1090 Vienna, Austria; 8 Leibniz-Institute of Surface Engineering (IOM), 04318 Leipzig, Germany; christian.laube@iom-leipzig.de; 9 Department of Clinical Pharmacology, Medical University of Vienna, 1090 Vienna, Austria; 10 Medical Imaging Cluster (MIC), Medical University of Vienna, 1090 Vienna, Austria

**Keywords:** nanodiamonds, small animal PET, preclinical imaging, AR42J tumor-bearing mice

## Abstract

Nanodiamonds (NDs) are emerging as a novel nanoparticle class with growing interest in medical applications. The surface coating of NDs can be modified by attaching binding ligands or imaging probes, turning them into multi-modal targeting agents. In this investigation, we assessed the targeting efficacy of octreotide-functionalized ^68^Ga-radiolabelled NDs for cancer imaging and compared it with the tumor uptake using [^68^Ga]Ga-DOTA-TOC. In vivo studies in mice bearing AR42J tumors demonstrated the highest accumulation of the radiolabeled functionalized NDs in the liver and spleen, with relatively low tumor uptake compared to [^68^Ga]Ga-DOTA-TOC. Our findings suggest that, within the scope of this study, functionalization did not enhance the tumor-targeting capabilities of NDs.

## 1. Introduction

Biomedical imaging facilitates the exploration of living organisms, offering molecular and anatomical insights into biological processes. Most imaging modalities rely on contrast agents or radiolabeled molecules as generic sources for the acquisition of image data with sufficient signal-to-noise quality. Among those contrast agents, polymeric and inorganic nanoparticles have been assessed for tumor imaging as these nanoparticles excerpt passive accumulation in tumors via the enhanced permeability and retention (EPR) effect. The EPR effect is intricately linked to the particle size, biocompatibility, and surface charge of the respective nanoparticle. Moreover, tumor vascularization and lymphatic drainage govern the retention of nanoparticles in target tissues, which consequently affects image contrast when such nanoparticles are used as generic imaging agents [1,2]. Even though the clinical utilization of the EPR effect remains nascent, its application in facilitating the passive accumulation of nanoparticles in tumors has been extensively evaluated for tumor imaging, nanomedicinal strategies, and nanoparticle-based therapies [3,4].

Among the diverse nanoparticles investigated as imaging probes, nanodiamonds (NDs) have emerged as promising tools for biomedical imaging applications [5,6]. Nanodiamonds, carbon-based nanoparticles ranging in size from 1 to 150 nm, possess distinctive physical and biomedical attributes. They demonstrate chemical inertness, inherent biocompatibility, and low toxicity [7]. Furthermore, NDs feature abundant optical color center defects, primarily attributed to nitrogen impurities during ND aggregation. These nitrogen-vacancy (NV) color defect centers serve as excellent fluorophores, exhibiting intense fluorescence emission without notable photobleaching. Notably, surface modifications of NDs have a minimal impact on their fluorescence properties. Consequently, NDs are amenable to various chemical modifications, facilitating their utilization in numerous fluorescence and photoacoustic imaging applications [8,9].

Additionally, NDs inherit intrinsic para- and diamagnetic properties, ultimately resulting in longitudinal relaxation time (*T*_1_)-enhancement when used as a contrast agent in magnetic resonance imaging (MRI) [10,11].

The surface modification of NDs can be achieved using various chemical strategies, typically involving biocompatible polymers or amino acids to enhance the colloidal stability and establish a platform for functionalization [12,13]. Targeting capabilities are introduced by functionalizing the ND surface with biomolecules that target receptors overexpressed on specific cells [14]. Furthermore, NDs display a high loading capacity, ultimately releasing high concentrations of potential payloads at the target moiety. Therefore, NDs provide significant theranostic potential when used as delivery agents for small molecule chemotherapeutics, peptides, or DNA/RNA [15,16]. Collectively, the inherent attributes of intrinsic and enduring photostable fluorescence, coupled with their capacity to enhance the *T*_1_ contrast in MRI applications, render ND-based contrast agents highly promising for diverse biomedical applications. Furthermore, their remarkable versatility in surface modifications and potential therapeutic payloads amplifies their potential for cross-scaled multimodal imaging and theranostics, spanning various dimensions of biological exploration.

In previous studies, we explored the potential of NDs for positron-emission tomo-graphy (PET) imaging [10,17]. In these studies, we coated NDs with cationized human serum albumin (cHSA) and polyethylene glycol (PEG) and added a chelator (DFO) for radiolabeling. The synthesis and radiolabeling of the cHSA-PEG-DFO-NDs were successful; however, the preclinical evaluation in tumor-bearing mice revealed a low tumor uptake. To improve the tumor uptake, a targeting agent functionalized on the surface coating of the NDs might be a promising strategy.

Hence, the present study explored the potential of radiolabeled coated NDs, functionalized with a somatostatin analog (octreotide; Oct) for specific tumor-targeted imaging. The tumor-accumulation capability of radiolabeled functionalized NDs was investigated and compared with [^68^Ga]Ga-DOTA-TOC as the “gold standard” in mice bearing a somatostatin overexpressing subcutaneous tumor.

## 2. Results

### 2.1. Radiolabeling of NDs

**Figure 1:**
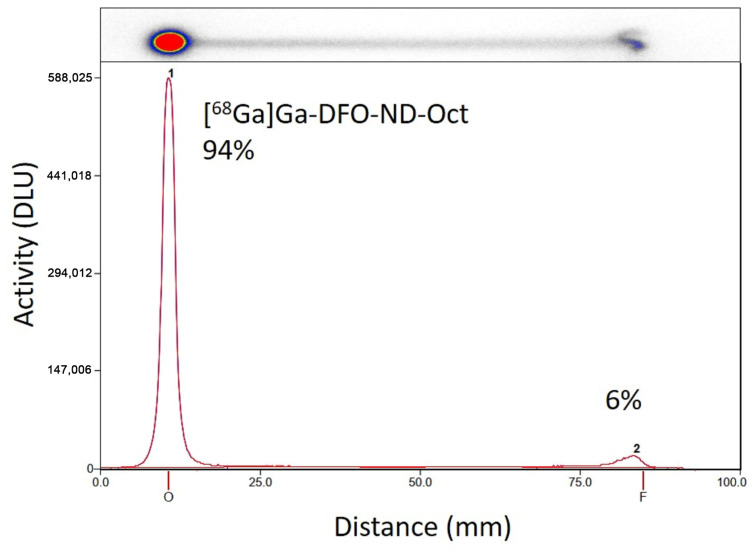
Radio TLC (silica gel RP18, citrate buffer pH 4.6) of [^68^Ga]Ga-DFO-ND-Oct radio-TLC quality control. Peak “1” — [^68^Ga]Ga-DFO-ND-Oct-product; Peak “2” — unconjugated [^68^Ga]Ga(citrate).

Radiolabeling of coated and functionalized NDs was achieved with a radiochemical purity of 91.1% ± 2.2% for [^68^Ga]Ga-DFO-ND-Oct (n = 4), respectively, within a total synthesis time of 60–80 min (for an example TLC analysis see Figure 1).

### 2.2. Biodistribution and PET Imaging of Radiolabeled Targeting NDs

**Figure 2:**
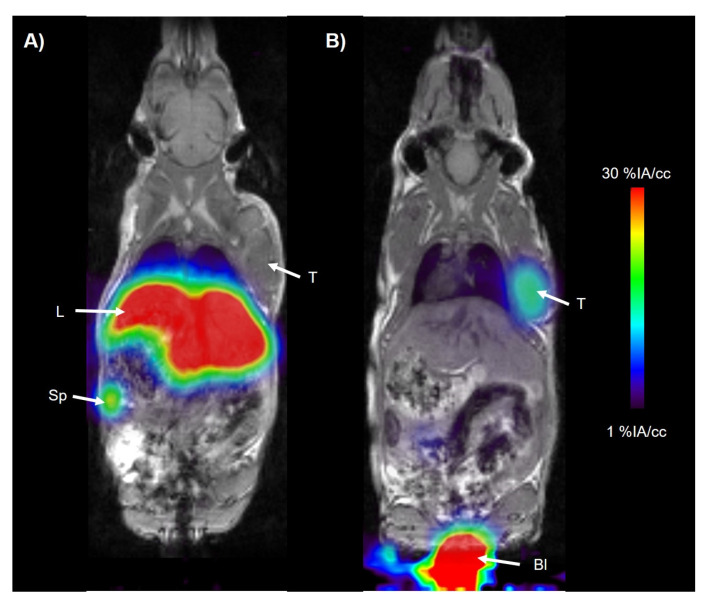
Representative horizontal co-registered PET/MR image showing the biodistribution of (**A**) [^68^Ga]Ga-DFO-ND-Oct and (**B**) [^68^Ga]Ga-DOTA-TOC in an AR42J tumor-bearing CD1 mouse. The radiation scale was set from 1 to 30%ID/cc. Organs of interest are indicated with arrows: Sp—spleen, L—liver, T—tumor, Bl—urinary bladder.

**Figure 3:**
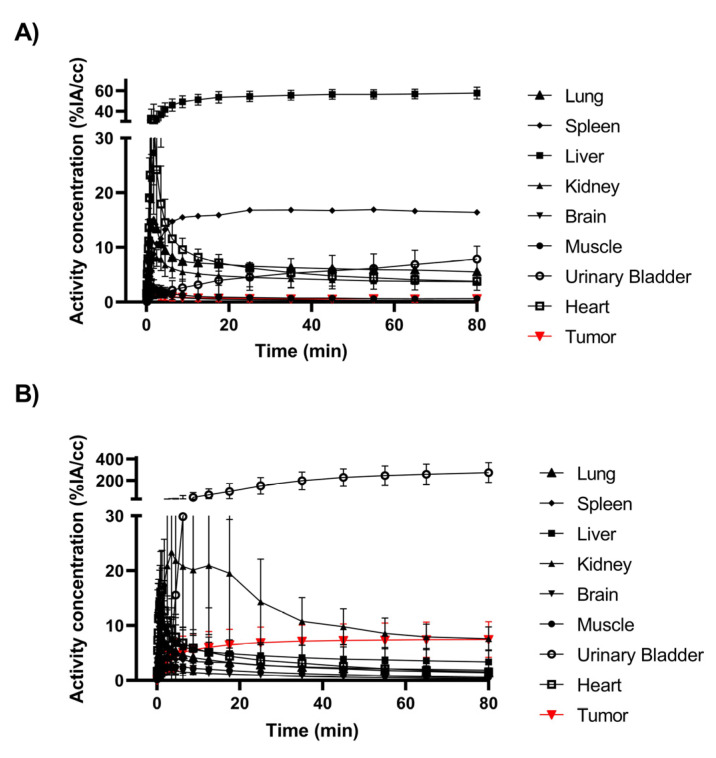
PET time–activity curves (TACs) obtained after injection of (**A**) [^68^Ga]Ga-DFO-ND-Oct (n = 6; %IA/cc) or (**B**) [^68^Ga]Ga-DOTA-TOC (n = 8; %IA/cc) in the AR42J tumor-bearing CD1 mice. The TACs show the mean value ± the standard deviation.

Dynamic PET imaging was performed for 90 min after injecting [^68^Ga]Ga-DFO-ND-Oct and [^68^Ga]Ga-DOTA-TOC. In Figure 2, PET/MR summation images illustrate the biodistribution of the tested compounds. The time–activity curves (TACs) obtained from the image analyses are depicted in Figure 3.

**Figure 4:**
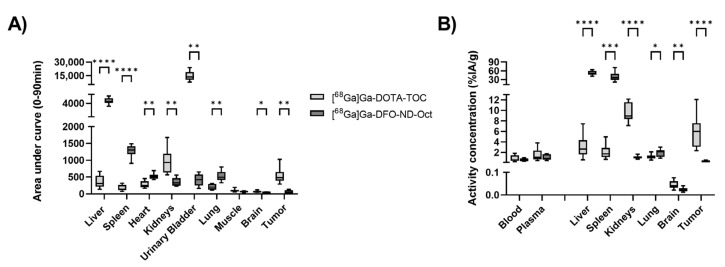
(**A**) Calculated area-under-the-curve from the 90 min PET for [^68^Ga]Ga-DFO-ND-Oct (n = 6) and [^68^Ga]Ga-DOTA-TOC (n = 8). In (**B**) the radioactivity values obtained from the biodistribution study (gamma-counter) at 95 min p.i. (%IA/g; n = 11/16) in AR42J tumor-bearing CD1 mice are shown. The box plot shows the mean value, and the whiskers indicate the 5–95 percentile. * *p* < 0.05, ** *p* < 0.01, *** *p* < 0.001, **** *p* < 0.0001; unpaired *t*-test with Welch correction.

Based on the TACs, the area-under-the curve values (AUCs) were calculated and are shown in Figure 4A. We obtained statistically significant differences between the [^68^Ga]Ga-DFO-ND-Oct and [^68^Ga]Ga-DOTA-TOC AUCs in nearly all analyzed organs except for the muscle. Figure 4B shows the results from the biodistribution study comparing activity concentrations between [^68^Ga]Ga-DFO-ND-Oct and [^68^Ga]Ga-DOTA-TOC. The comparison of the [^68^Ga]Ga-DFO-ND-Oct activity concentration with [^68^Ga]Ga-DOTA-TOC did reveal statistically significant differences in all analyzed organs and tumors except blood and plasma. The values from the gamma counter are summarized in Table 1. In addition, the values from the previous study focusing on [^68^Ga]Ga-DFO-ND [17] are shown in the table.

When comparing the biodistribution values between [^68^Ga]Ga-DFO-ND-Oct and [^68^Ga]Ga-DFO-ND, the two-sided unpaired *t*-test revealed compelling biological evidence of divergence in the kidney (t(20) = 3.48, *p* = 0.002, d = 1.5) and brain (t(20) = 2.38, *p* = 0.027, d = 1.01) between these two groups. For the other organs, we observed a reduction in the [^68^Ga]Ga-DFO-ND-Oct radioactivity concentration in blood, plasma, tumor, and spleen, and an increase in liver and lung compared to [^68^Ga]Ga-DFO-ND, although these differences did not reach statistical significance.

## 3. Discussion

Radiolabeled nanodiamonds have emerged as innovative drug carriers with enhanced biocompatibility, a point underscored by the editorial board of the EJNMMI Radiopharmacy and Chemistry journal [18]. In a prior investigation, we developed a protocol for coating and radiolabeling nanodiamonds (NDs), assessing their in vivo pharmacokinetics in mice with tumors [17]. Due to the initially low tumor uptake, we devised strategies to enhance the tumor uptake, one of which involved functionalizing the NDs by incorporating a tumor-targeting moiety. Opting for octreotide, a well-established somatostatin receptor-targeting radiopharmaceutical in clinical use as a preclinical study model [19,20], we employed the trans-cyclooctene (TCO) tetrazine click-chemistry approach [21] to integrate octreotide into the NDs’ surface coating. Subsequently, we evaluated the feasibility of using ^68^Ga-radiolabeled NDs functionalized with octreotide for tumor imaging, comparing it with the established [^68^Ga]Ga-DOTA-TOC “gold standard”. However, our efforts failed to reveal enhanced tumor-targeting capabilities with functionalized [^68^Ga]Ga-DFO-ND-Oct.

In a previous study [17], we explored the biodistribution of non-functionalized [^68^Ga]Ga-DFO-ND. Despite including a targeting moiety in the present study, we did not observe increased ND uptake in the tumor compared to non-functionalized NDs ([^68^Ga]Ga-DFO-ND: tumor 0.37 ± 0.10 %IA/g [17] versus [^68^Ga]Ga-DFO-ND-Oct: tumor 0.32 ± 0.12 %IA/g). Moreover, the [^68^Ga]Ga-DFO-ND-Oct group exhibited a reduced kidney and blood concentration and increased liver uptake compared to the [^68^Ga]Ga-DFO-ND group (see Table 1). Although not all observed differences reached statistical significance, we attribute this to the slightly larger size (156 nm) of [^68^Ga]Ga-DFO-ND-Oct compared to [^68^Ga]Ga-DFO-ND (146 nm). This finding aligns with a previous investigation that utilized ^125^I-labeled bovine serum albumin (BSA) covalently attached to fractionated detonation nanodiamonds (DNDs) of varying sizes [22]. The DND-BSA^I125^ samples, with average particle sizes of 62 nm, 181 nm, and 266 nm, were administered intravenously to adult male mice. A biodistribution analysis conducted 1 h after intravenous administration revealed a decreased kidney and blood concentration and increased liver uptake with larger particle sizes. In another study by Hirn et al. [23], gold nanoparticles of five different sizes (1.4 nm, 5 nm, 18 nm, 80 nm, and 200 nm) were administered via i.v. injection into female Wistar-Kyoto rats. Samples (organs, blood, and excretion) were collected after 24 h. They showed a strong size dependency on the distribution and accumulation of gold NPs in all organs, tissues, and excretion. Hence, the uptake pattern of nanoparticles is strongly influenced by their size.

Furthermore, the uptake of nanodiamonds (NDs) is significantly influenced by their surface coating and charge. Rawal et al. [24] noted that various physicochemical properties, such as particle size, shape, polydispersity, and surface charges, play crucial roles in determining the safety, efficacy, pharmacokinetics, pharmacodynamics, and biodistribution of nanomaterials. Positively charged particles tend to be captured by macrophages in organs such as the lungs, liver, and spleen, while neutral or slightly negatively charged nanoparticles exhibit longer circulation times and reduced accumulation in these organs. Additionally, Xiao et al. [25] demonstrated that positively charged PEG-oligocholic acid-based micellar nanoparticles (NPs) with a 15–20 nm size range exhibited higher liver uptake in nude mice with SKOV-3 human ovarian cancer xenografts. Conversely, nanoparticles with slightly negative surface charges showed very low liver uptake but high tumor uptake. Hirn et al. [23] further demonstrated that nanoparticles with a positive charge exhibit increased susceptibility to clearance via the hepato-biliary pathway.

Moreover, both the size and surface charge play significant roles in influencing the EPR effect [26,27,28]. Studies have demonstrated that NPs falling within the 100–200 nm size range are considered optimal for exploiting the EPR effect in solid tumors [29], a range that encompasses our current NDs. However, it has also been observed that NPs with high positive charges are prone to being captured and retained by the vascular endothelial luminal, which is rich in negatively charged phospholipids. Conversely, nanoparticles with elevated negative charges tend to be absorbed and cleared by organs such as the liver, spleen, or other components of the reticuloendothelial system (RES). Consequently, the ideal surface charge for NPs should be neutral or slightly negative [30]. In our current investigation, the coated NDs bore a positive surface charge, which may have contributed to the relatively low tumor uptake observed.

In addition, it also has to be mentioned that the administered activities and injected masses were different between the [^68^Ga]Ga-DFO-ND and [^68^Ga]Ga-DOTA-TOC groups, potentially biasing the obtained results.

Furthermore, it remains unclear whether the affinity of octreotide was diminished by the tetrazine-TCO ligation and the presence of the nanodiamonds (NDs). Reubi et al. [31] highlighted that even minor structural alterations, chelator substitutions, or metal replacements can significantly impact the binding affinity of somatostatin radioligands. Hence, assessing the somatostatin receptor subtype affinity profile of the functionalized NDs becomes crucial. However, conventional in vitro binding assays face a challenge as separating unbound NDs from cells proves impractical. Moreover, determining peptide concentrations and molar extinction coefficients poses another challenge. Quantifying peptide concentrations relies on knowledge about the exact elemental composition of the octreotide complex and its known peptide content. Given that we introduced octreotide via the tetrazine-TCO click reaction, resulting in cHSA-PEG-DFO-TCO-Tz-Oct as the coating material, determining the precise amount of Oct per ND was unfeasible. However, it is important to note that the absence of specific binding in vitro data represents a limitation of the present study. Hence, generating in vitro data is advisable for subsequent investigations to discern whether the in vivo ineffectiveness of ND-Oct for tumor targeting stems from inadequate active accumulation or passive uptake.

## 4. Materials and Methods

### 4.1. Chemicals and Radiotracer

The following materials were used for the cell culture: PBS, 0.25% Trypsin-EDTA, FBS, Penicillin/Streptomycin, RPMI 1640, and L-glutamine, and were obtained from Gibco (Fisher Scientific, Vienna, Austria).

[^68^Ga]Ga-DOTA-TOC was synthesized with slight modifications of the described production method [32,33]. Briefly, the 30 mCi (1110 MBq) ^68^Ge/^68^Ga radionuclide generator (Cyclotron Co., Ltd., Obninsk, Russia) was eluted using 5 mL 0.1 M HCl and the middle fraction (130 MBq in 1 mL) was used. To 800 µL ^68^Ga-Cl_3_ eluent, 160 µL 0.25 M Na_2_CO_3_ was added to adjust the pH to 5.5–6.5. Then, 50 µg DOTA-TOC in 200 µL acetate puffer (1.5 M, pH 3.8) was added and reacted for 7 min at 100° C and 600 rpm in a thermomixer (Eppendorf, Hamburg, Germany). Uncomplexed ^68^Ga was removed through retention on a reversed-phase cartridge (tC18 SepPAK; Waters Corp., Milford, MA, USA), whereas [^68^Ga]Ga-DOTA-TOC was eluted with ethanol (1 mL). After evaporation of the organic solvent, the compound was redissolved in 600 µL PBS. Radiochemical yield was 78 ± 15% (n = 5) with >99% of the radioactivity migrated with an Rf ~0.6 corresponding to [^68^Ga]Ga-DOTA-TOC as assessed using radio-HPLC. The molar activity was 12 ± 6 GBq/µmol at the end of synthesis.

### 4.2. Radiolabelling of NDs

Cleaned NDs (MSY 0–0.2) with an averaged diameter of 100 nm (calculated from transmission electron microscopy) were provided by Microdiamants AG (Lengwil, Switzerland) and pretreated as described previously [34]. A biocompatible, non-covalent coating strategy was developed to stabilize NDs in biological media and to render functionalization more efficient and reproducible. The surface coating preparation for the NDs was performed following previously described procedures [35]. In brief, human serum albumin was cationized to achieve cHSA and then stabilized by the addition of PEG polymer chains yielding cHSA-PEG. Then, p-SCN-Bn-Deferoxamine (also known as desferal, DFO; 752 g/mol) was introduced to cHSA-PEG by reacting the thiocyanate of DFO with free primary amine groups of cHSA-PEG. After the removal of unreacted DFO on the next day, PEG chains containing a trans-cyclooctene group (PEG-TCO; 867 g/mol) were introduced to cHSA-PEG-DFO. Finally, free PEG-TCO was removed by ultrafiltration, yielding cHSA-PEG-DFO-TCO. To attach the targeting group octreotide (Oct) to cHSA-PEG-DFO-TCO via click chemistry, octreotide was site-selectively modified with a tetrazine-PEG rebridging agent, according to a previous publication [36]. In short note, octreotide contains a single disulfide bond which, after cleavage of this disulfide bond through the addition of reducing agents, could be rebridged using an allyl sulfone reagent yielding octreotide–tetrazine (Tz-Oct). The final product was characterized using electrospray ionization–mass spectrometry and liquid chromatography–mass spectrometry. Subsequently, 0.5 equivalents of octreotide-tetrazine (Tz-Oct) were added to a solution of cHSA-PEG-DFO-TCO in 1 mL phosphate buffer (50 mM, pH 8.0) at room temperature for 2 h. After free octreotide–tetrazine was removed using ultrafiltration (MWCO: 30 kDa), MALDI-TOF mass spectrometry revealed the introduction of 8 units of octreotide yielding cHSA-PEG-DFO-TCO-Tz-Oct (148 kDa).

NDs were coated with cHSA-PEG-DFO-TCO-Tz-Oct according to the literature [35]. In brief, NDs were diluted to low concentration (0.1 mg/mL) in MilliQ water, and afterward, a solution of cHSA-PEG-DFO-TCO-Tz-Oct (0.1 mg/mL, 4 times mass excess) in MilliQ water was dropped into the ND solution and the mixture was stirred overnight. The free proteins were removed using centrifugation the following day, yielding ND-cHSA-PEG-DFO-TCO-Tz-Oct (DFO-ND-Oct). The successful coating was confirmed using dynamic light scattering (DLS). The average diameter increased from 138 ± 0.5 nm for uncoated NDs to 156 ± 3.8 nm for DFO-ND-Oct. The polydispersity index (PDI) did increase from 0.1 ± 0.01 (uncoated NDs) to 0.4 ± 0.05 for the DFO-ND-Oct and the surface charge changed from a negative value for the uncoated NDs (~−40 mV) to around 30 mV after coating [37]. Radiolabeling was performed as described previously [17]. In brief, prior to radiolabeling, a ^68^Ge/^68^Ga radionuclide generator (Cyclotron Co., Ltd., Obninsk, Russia) was fractionally eluted using 0.1 M HCl. For labeling, 0.2 mL of the middle fraction (18.1 ± 5.6 MBq) was used, and the pH was adjusted to 5.0–6.5 using 0.25 M aq. Na_2_CO_3_ solution. Before radiolabeling, the DFO-ND-Oct (0.5 mg/mL in H_2_O) were dispersed in an ultrasonic bath or a rotary vortex for 15 min. To the pH-adjusted ^68^Ga-solution, 0.45 mL DFO-ND-Oct (0.22 mg) was added and mixed for 60 min in an overhead shaker (Grant Instruments (Cambridge) Ltd., Shepreth, UK) using the following parameters: orbital: 35 rpm for 5 s, reciprocal: 90° for 10 s, vibro/pause: 5/5 s. Quality control was performed using thin-layer chromatography (TLC) using 0.1 M citrate buffer at pH 4.6 as mobile phase. Samples from the radiolabeled ND solution and ^68^Ga-Cl_3_ solution as control were spotted on silica gel RP 18 TLC plates (2.5 × 10 cm; Merck, Darmstadt, Germany), and plates were developed. Detection was performed by placing the TLC plates on multi-sensitive phosphor screens (Perkin-Elmer, Rodgau, Germany). Radiolabeling efficiency was calculated based on the peaks at the start and front, assuming that the radiolabeled NDs would remain at the start position.

### 4.3. Animal Model

The rat pancreatic carcinoma cell line AR42J (ECACC 93100618) was purchased from ECACC (Public Health England Culture Collections, Salisbury, UK). This cell line was chosen for the tumor targeting experiments as it was shown to express the highest somatostatin receptor subtype 2 (SSTR2) concentration [38]. AR42J cells were cultured in CytoOne (Starlab) flasks containing RPMI 1640 medium supplemented with 2 nM L-Glutamine plus 10% FBS and antibiotics (100 IU penicillin + 0.1 mg/mL streptomycin). Cells were maintained at 37 °C under a humified condition of 95% air and 5% CO_2_ and passaged once weekly before experiment use.

Female Crl:CD1-Foxn1nu mice (Charles River Laboratories, Sulzfeld, Germany) aged 7–8 weeks were used (n = 27, 26.3 ± 2.2 g). Animals were housed in groups (4–6 animals) in polysulfone type III cages under individual ventilated cage conditions in a temperature and humidity-controlled facility (22 ± 3 °C; 40% to 70% humidity), had free access to standard laboratory animal diet (ssniff R/M-H, ssniff Spezialdiäten GmbH, Soest, Germany) and water ad libitum, and were kept under a cycle of 12 h of light and 12 h of dark. An acclimatization period of at least 1 week was allowed before the animals were used in the experiments. Then, animals were anesthetized in an induction box with isoflurane in air, and 5 × 10^6^ AR42J cells in a volume of 100 µL PBS were injected subcutaneously in the right shoulder region of nu/nu CD1 mice. Tumor sizes were measured once per week using a caliper, and around 16 days after inoculation, when tumors had reached a size of 1480 ± 970 mm^3^ (range 140–3870 mm^3^), animals were used in the imaging experiments.

The Amt der Niederösterreichischen Landesregierung approved the studies in compliance with the Austrian Laboratory Animal Experimentation Act) and study procedures were in accordance with the European Communities Council Directive of 22 September 2010 (2010/63/EU).

### 4.4. PET/MR Imaging and Biodistribution

AR42J tumor-bearing animals were divided into 2 groups for the imaging and biodistribution experiments. Before each experiment, the animals were placed in an induction box and anesthetized with ~3.5% isoflurane in air. When unconscious, the animals were taken from the induction box and positioned on the heated double-imaging chamber for PET imaging (n = 6–8/group) or on a heated mat for ex vivo gamma counting (n = 5–8/group) in prone position, and anesthesia was continued. For PET imaging, a microPET Focus 220 scanner (Siemens Medical Solutions, Knoxville, TN, USA) was used [39]. Before radiotracer injection, a 10 min transmission scan using a rotating ^57^Co source was recorded. An energy window of 250–750 keV and a timing window of 6 ns were used to acquire the dynamic PET scans. During the scans, animal respiratory rate and body temperature were constantly monitored (SA Instruments Inc., Stony Brook, NY, USA), and the isoflurane level was adjusted (1.5–2.5% in oxygen) to achieve a constant depth of anesthesia. The tails were warmed, and a catheter was positioned into the lateral tail vein for radiotracer injection. [^68^Ga]Ga-DFO-ND-Oct (n = 11, 1.6 ± 0.4 MBq, 200 µL, 62.5 ± 0.1 µg NDs) or [^68^Ga]Ga-DOTA-TOC (n = 16, 8.3 ± 1.7 MBq, 150 µL, 1.6 ± 1.1 µg) was injected and a 90 min PET scan or 90 min uptake period was started. Some animals additionally underwent a T1-FLASH (TE: 7 ms, TR: 30 ms, rep.: 5, FOV (mm): 76 × 28 × 24, matrix: 217 × 80 × 34, resolution (cm/pixel): 0.35 × 0.35 × 0.70, orientation: coronal, scan time: 8 min, flip angle: 35°) weighted MRI scan (1T ICON scanner, Bruker, Ettlingen, Germany) directly before the PET scan. At 90 min after injection, blood was collected using puncture of the retrobulbar plexus under anesthesia. Animals were sacrificed, and organs (brain, spleen, kidneys, liver, lung) and tumors were sampled from all animals (n = 11(16)/group) and measured in the gamma counter. The animal number, weight, injected activity, and number of animals per group are summarized in Table 2. Imaging method descriptions follow Stout et al.’s guidelines [40].

### 4.5. Ex Vivo Analysis of Samples

Blood was centrifuged to obtain plasma (17,000× *g*, 4 °C, 1 min), and aliquots of blood and plasma were transferred into pre-weighted test tubes. Radioactivity concentration in the organ samples was measured in a gamma counter (HIDEX AMG Automatic Gamma Counter, Turku, Finland). Empty and full tubes were weighted to obtain organ weight. The gamma counter was calibrated using a series of tubes with decreasing activity of a ^68^Ga-solution.

Data from the gamma counter expressed in kBq/g were decay-corrected to the time of radiotracer injection. Then, data points were corrected using the injected activity and displayed as percent injected activity per gram tissue (%IA/g).

### 4.6. PET/MR Image Data Analysis

Dynamic PET emission data were sorted into 24 frames, which incrementally increased in time length from 5 s to 20 min. All PET images were reconstructed using Fourier rebinning of the 3D sinograms followed by two-dimensional filtered back projection with a ramp filter, resulting in a voxel size of 0.4 × 0.4 × 0.798 mm^3^. The standard data correction protocol (normalization, attenuation, and decay correction) was applied to the PET data. The PET units were converted into units of radioactivity by applying a calibration factor yielding kBq/cc. After that, the values were corrected using injected activity and expressed as %IA/cc. PET images were analyzed using the image analysis software AMIDE version 1.0.4 42 [41]. The MR image was co-registered with the PET image using rigid transformation, and volumes of interest (VOI) covering the whole tumor area were manually drawn. In addition, predefined spherical VOIs were drawn over the brain, muscle, liver, spleen, kidneys, urinary bladder, lung, and heart on the PET time frame where the organs were clearly visible. Afterward, the PET time–activity curves (TACs) for each organ and tumor were extracted. The area-under-the-curve (AUC) of the PET TACs from 0 to 90 min was calculated for the different VOIs.

### 4.7. Statistics

Statistical testing was performed using GraphPad Prism 9.5.1 software (GraphPad Software, La Jolla, CA, USA). Differences between the groups were analyzed using a 2-sided unpaired *t*-test with Welch correction using the Holm–Sidak method and assuming individual variance for each group. The level of statistical significance was set to *p* < 0.05. Unless stated otherwise, all values are given as mean ± standard deviation (SD).

## 5. Conclusions

Our findings underscore the practical limitations of the current functionalized NDs in terms of biodistribution within our conducted studies. These results serve as a foundation for future enhancements in ND characteristics, focusing on aspects such as size, surface coating, coating modification, and targeted specificity. 

## Figures and Tables

**Table 1:** Radioactivity values obtained from the biodistribution study (gamma counter) at 95 min p.i. in AR42J tumor-bearing CD1 mice.

	[^68^Ga]Ga-DFO-ND-Oct %IA/g	[^68^Ga]Ga-DOTA-TOC %IA/g	[^68^Ga]Ga-DFO-ND [17] %IA/g
n	11	16	11
Blood	0.57 ± 0.26	0.78 ± 0.54	0.93 ± 0.61
Plasma	1.01 ± 0.49	1.46 ± 1.05	1.76 ± 1.16
Tumor	0.32 ± 0.12	5.38 ± 2.98	0.37 ± 0.10
Spleen	40.52 ± 15.36	2.00 ± 1.21	41.26 ± 13.28
Liver	52.47 ± 7.76	2.99 ± 2.01	47.75 ± 9.31
Kidneys	1.09 ± 0.28 #	9.25± 1.95	1.45 ± 0.19 # (*p* = 0.002)
Lung	1.94 ± 0.70	1.31 ± 0.96	1.89 ± 0.38
Brain	0.02 ± 0.01 #	0.04 ± 0.02	0.04 ± 0.02 # (*p* = 0.027)

# 2-sided unpaired *t*-test with Welch correction using the Holm–Sidak method.

**Table 2:** Overview of animal weight, injected activity, mass, and the number of animals used in the experiments.

	[^68^Ga]Ga-DFO-ND-Oct	[^68^Ga]Ga-DOTA-TOC
Body weight [g]	25.2 ± 1.7	27.1 ± 2.4
Injected activity [MBq]	1.6 ± 0.4	8.3 ± 1.7
Injected mass [µg]	62.5 ± 0.1	1.6 ± 1.1
PET imaging, n	6	8
Biodistribution, n	5	8

## Data Availability

The data presented in this study are available on request from the corresponding author.
